# Frontiers of Medical Micro/Nanorobotics: *in vivo* Applications and Commercialization Perspectives Toward Clinical Uses

**DOI:** 10.3389/fbioe.2018.00170

**Published:** 2018-11-14

**Authors:** Fernando Soto, Robert Chrostowski

**Affiliations:** ^1^Department of Nanoengineering, University of California, San Diego, La Jolla, CA, United States; ^2^Texas Materials Institute, The University of Texas at Austin, Austin, TX, United States

**Keywords:** nanomedicine, medical translational research, *in vivo*, commercialization, microrobot, nanorobot

## Abstract

The field of medical micro/nanorobotics holds considerable promise for advancing medical diagnosis and treatment due to their unique ability to move and perform complex task at small scales. Nevertheless, the grand challenge of the field remains in its successful translation towards widespread patient use. We critically address the frontiers of the current methodologies for *in vivo* applications and discuss the current and foreseeable perspectives of their commercialization. Although no “killer application” that would catalyze rapid commercialization has yet emerged, recent engineering breakthroughs have led to the successful *in vivo* operation of medical micro/nanorobots. We also highlight how standardizing report summaries of micro/nanorobotics is essential not only for increasing the quality of research but also for minimizing investment risk in their potential commercialization. We review current patents and commercialization efforts based on emerging proof-of-concept applications. We expect to inspire future research efforts in the field of micro/nanorobotics toward future medical diagnosis and treatment.

## Introduction

Imagine a world where robots the size of cells operate inside our body. This might sound like a science fiction story written by Isaac Asimov, or a visionary speech from Richard Feynman; however, it is conceivable that micro/nanorobotics will soon play a prominent role in medicine. (Wang, 2013; Wang et al., 2013; Li et al., 2017b) We use the term medical micro/nanorobots to refer to all nano- to micron-size structures (300 nm−300 μm) capable of converting power sources into kinetic energy.Three groups of powered micro/nanorobots are mainly described. Biohybrid systems integrate synthetic nanostructures with motile microorganisms as the engine of the micro/nanorobot. (Kim and Tung, 2015; Ricotti et al., 2017; Bente et al., 2018; Palagi and Fischer, 2018). Chemically powered micro/nanorobots use asymmetric catalytic engines to selectively convert chemical fuels into locomotion (Chen et al., 2016; Nourhani et al., 2017; Schattling et al., 2017). Physically powered nanorobots convert external energy inputs (e.g., magnetic, ultrasound, or light fields) into translational motion based on engine geometry and material designs (Garcia-Gradilla et al., 2013; Dai et al., 2016; Bi et al., 2018; Pal et al., 2018).

**Figure 1:**
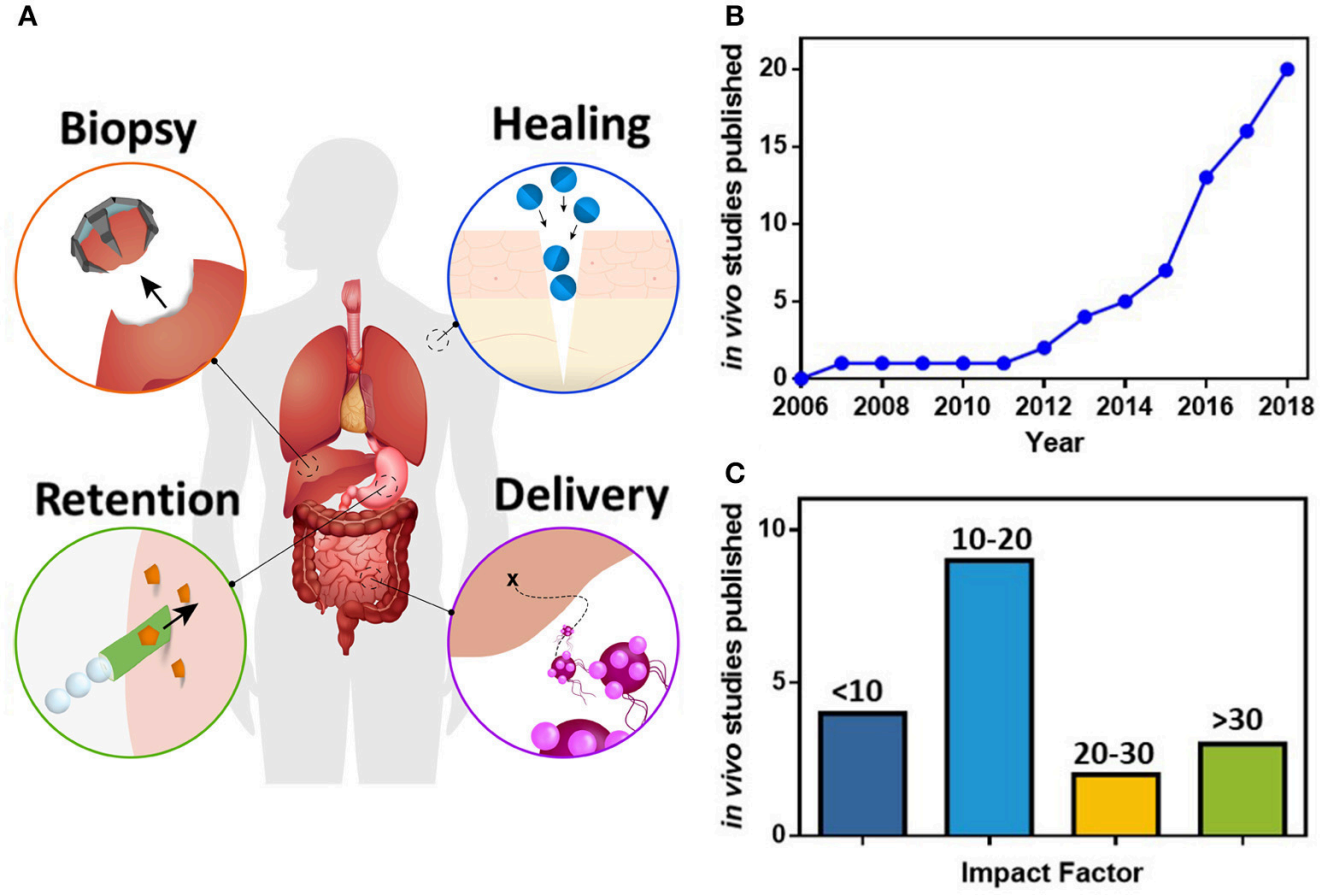
**(A)** Scheme of medical perspectives in micro/nanorobotics for *in vivo* human applications, including machines capable of performing biopsy (Gultepe et al., 2012), healing wounds (He et al., 2016), enhanced retention in tissues (Gao et al., 2015), and deliver their cargoes to specific destinations (Felfoul et al., 2016). Analysis of the published articles (see Table 1), including micro/nanorobots for *in vivo* applications, showing **(B)** the cumulative number of published articles, and **(C)** the impact factor of those publications.

The ability of micro/nanorobots to perform different task has been demonstrated at the laboratory scale, reporting their use for diverse proof of concept applications, including targeted cargo delivery (Solovev et al., 2010; Srivastava et al., 2018), fluid mixing (Orozco et al., 2013; Singh et al., 2015) and physical manipulation of micro objects (Cappelleri et al., 2014; Schuerle et al., 2017). The clinical aspirations of medical micro/nanorobots are still beyond the current capabilities of nanotechnology and bioengineering. Nevertheless, recent engineering breakthroughs have led to the successful *in vivo* operation of medical micro/nanorobots, illustrating initial proofs of concept for biopsy, delivery, healing and retention, as represented by the scheme in Figure 1A. While promising, these technological innovations are still challenging to translate into actual clinical therapies due to the safety concerns and the complexity of operating inside the human body.

This review focuses on the recent progress in the *in vivo* usage of micro/nanorobotics and on the efforts to commercialize and translate laboratory results into clinical applications. While there are several micro/nanorobot reviews addressing power and actuation principles (Sánchez et al., 2014; Teo and Pumera, 2016; Tu et al., 2017; Ren et al., 2018), fabrication procedures (Lin et al., 2015; Wang and Pumera, 2015; Jurado-Sánchez et al., 2017), and applications (Guix et al., 2014; Peng et al., 2017; Kim et al., 2018; Luo et al., 2018; Safdar et al., 2018), none of these reviews have addressed the crucial emerging clinical translations and potential commercial uses. We envision medical micro/nanorobotics as the frontier in treatment and diagnosis, potentially entailing benefits to human health by opening new therapies that are otherwise impossible to achieve.

## *In vivo* micro/nanorobotic applications

The applications of micro/nanorobots for medical purposes in animal models are still limited compared to the large number of *in vitro* proofs of concept. However, the increase in the number of cumulative *in vivo* micro/nanorobotic publications (Figure 1B) and the high impact factor of the journals in which they are published (Figure 1C), both attest to the advancement in medical applications for micro/nanorobots, and to the encouraging level of interest within the scientific community (based on reviewed articles summarized in Table 1). Although there are multiple methodologies to power micro/nanorobots, we identify that only biohybrid (20%), chemical (30%), and physical systems (50%) have been used within inside living animals. The *in vivo* studies are detailed in the following sections based on their clinical aspiration or area of study.

**Table 1:** *In-vivo* applications of micro/nanorobots divided into the power source, robotic design, animal model, and function.

**Power source**	**Robotic design (dimension)**	***In vivo* animal model (location)**	**Function**	**References**
Biohybrid	*L. monocytogenes* streptavidin-polystyreneNP (1 μm)	Mouse(intra-peritoneal cavity)	Targeted payload delivery for monitoring gene expression using fluorescence imaging	Akin et al., 2007
	*S. typhimurium* funtionalzied μ-particle (3 μm)	Mouse(circulatory system, thigh, tail vein).	Imaging of tumor site using fluorescence imaging	Park et al., 2013
	*S. Typhimurium* Engineered bacteria (1.2 μm)	Mouse(colon)	Controlled delivery and localized production of α-emolysin E (pore-forming toxin) against tumor	Din et al., 2016
	*Magnetococcus marinu*/MC-1 receptor (2 μm)	Mouse(peritumoral region)	Targeting hypoxic tumor regions	Felfoul et al., 2016
Chemical	Zn microrocket (15 μm)	Mouse (stomach)	Retention of cargo in the stomach	Gao et al., 2015
	CaCO_3_ Janus NP (10 μm)	Mouse (tail, liver)Pig (femoral artery)	Stop bleeding	Baylis et al., 2015
	Mg microrocket/enteric coating (15 μm)	Mouse(gastrointestinal tract)	Targeted retention of cargo in different parts of the gastrointestinal tract	Li et al., 2016
	Mg /Au /enteric coating Janus (20 μm)	Mouse (stomach)	Temporal neutralization of gastric acid and triggered payload release	Li et al., 2017b
	Mg TiO_2_ Janus NP (20 μm)	Mouse (stomach)	Pill to deliver large amount of microrobots	Karshalev et al., 2018
	Mg/TiO_2_/Chitosan Janus NP (20 μm)	Mouse (stomach)	*H. pylori* infection targeted therapy	Esteban-Fernández de Ávila et al., 2017b
Physical	Polymeric griper (300 μm)	Pig(biliary tree, bile duct)	Tissue biopsy	Gultepe et al., 2012
	Magnetic microrod (300 μm diameter)	Rabbit(eye)	Intraocular navigation	Ullrich et al., 2013; Pokki et al., 2016
	Ni Magnetic rod (300 nm x 2 μm)	Mouse (femoral vessels, brain)	Acceleration of thrombolysis	Cheng et al., 2014 Hu et al., 2018
	Helical structures (20 μm)	Mouse (intra-peritoneal cavity)	Controlled navigation and localization using optical imaging	Servant et al., 2015
	PEM–magnetite–gold Janus NP 5 μm	Mouse(skin)	Infrared laser-assisted tissue welding	He et al., 2016
	Spirulina microalgae magnetized 100 nm Fe_3_O_4_	Mouse (subcutaneous tissue, intraperitoneal cavity, stomach)	Controlled navigation and localization using optical and magnetic imaging	Yan et al., 2017
	FePd nanorod (300 nm x 4 μm)	Mouse(subcutaneous tissue)	Targeted delivery and triggered activation of fluorouracil	Hoop et al., 2018
	Burr-like porous sphere (50 μm)	Mouse(left dorsum)	Transport and delivery of cell cultures	Li et al., 2018

### Delivery of therapeutic and imaging agents for cancer therapy

Medical micro/nanorobotics hold great potential to deliver drugs with a higher degree of precision and speed when compared to passive diffusion methods. In general, this direction has been the motivation for *in vivo* applications (Erkoc et al., 2018). Thus, targeted delivery has benefited by recent *in vitro* developments in micro/nanorobotic chemotaxis (Peng et al., 2015; Shao et al., 2017) and material research using stimuli triggered drug release (Genchi et al., 2017; Rao et al., 2018). For example, magnetically guided nanorobots were used toward the delivery of fluorouracil medication for reducing tumor growth in a mice model. The released drug was externally triggered, allowing the nanorobotic platform to distribute a high amount of the therapeutic agent in a localized area of the tumor (Hoop et al., 2018).

**Figure 2:**
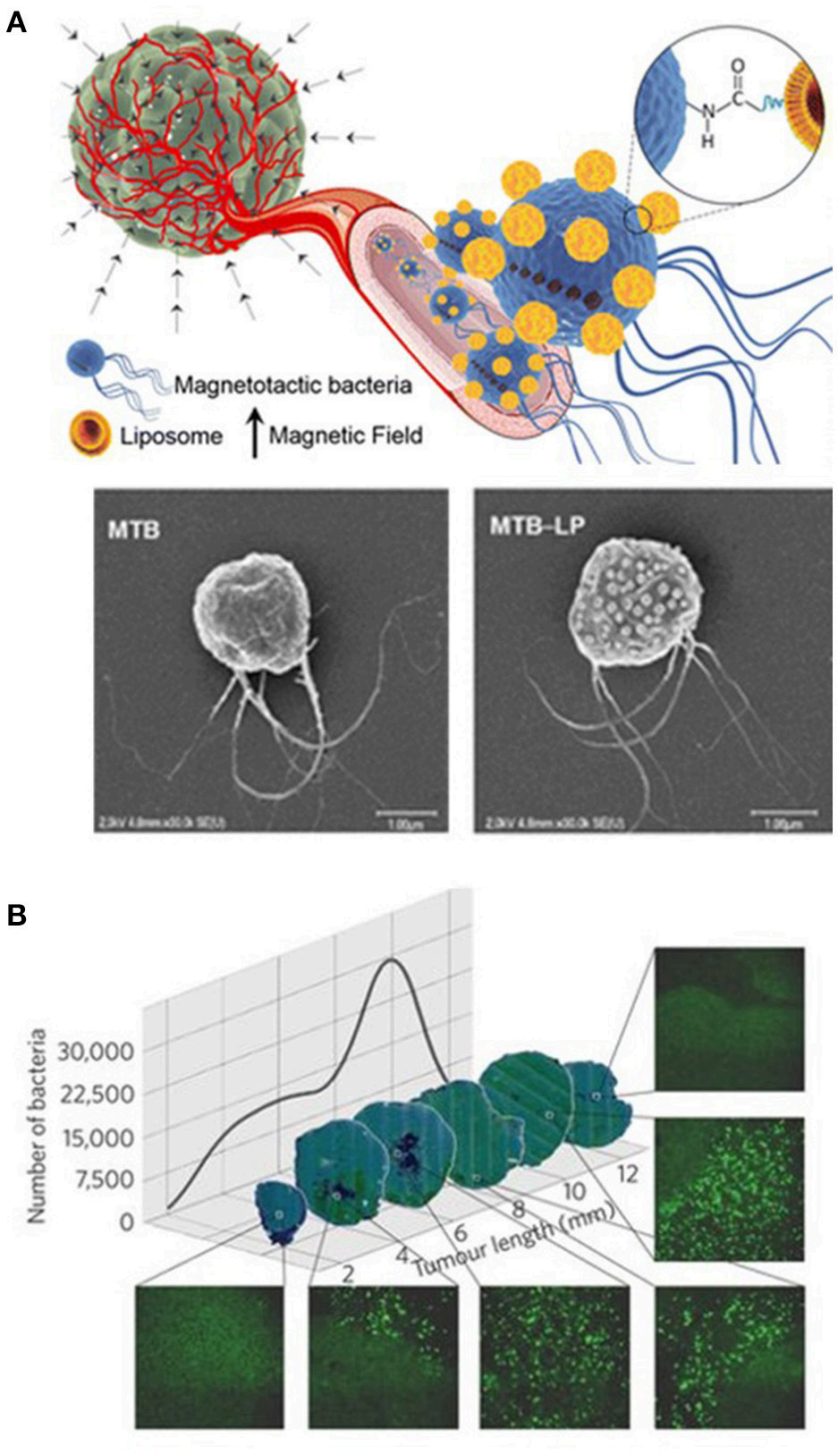
Nanorobots for delivery usage. **(A)** Schematic illustration and SEM of biohybrid nanorobot including a magnetotactic bacteria loaded with liposomes. Reprinted with permission from Taherkhani et al. (2014). Copyright 2014 American Chemical Society. **(B)** Fluorescent images of transverse tumor sections illustrating the biohybrid robot distribution and population inside the tumor. Reprinted with permission from Felfoul et al. (2016). Copyright 2016 Springer Nature.

Biohybrid nanorobots have also been used for targeted delivery of payloads inside living animals. *Listeria monocytogenes* has been used to deliver attached nanoparticles containing a payload of genes and proteins within a mouse. These payloads were used to monitor gene expression through differences in the luminescence produced within the different mouse organs (Akin et al., 2007). Magnetotactic bacterias, which naturally produce magnetic iron oxide nanoparticles, have been coupled with liposomes loaded with therapeutic payloads *in vitro*, as shown in Figure 2A (Taherkhani et al., 2014). More recently, these modified bacteria were guided using an external magnetic field to deliver the drug-loaded liposomes *in vivo* to a mouse tumor site (Figure 2B) (Felfoul et al., 2016).

The use of fully bio-engineered biohybrid micro/nanorobots without any inorganic/artificial components for carrying and transporting the therapeutic cargo has become possible through recent advances in synthetic biology. The use of genetically engineered bacteria, *S. Typhimurium*, have been reported to locally produce a therapeutic payload (α-emolysin E, a pore-forming toxin) and to trigger the payload's release upon bacterial lysis. A small number of bacteria survive the lysis event, which allows for a continuous and cyclical delivery process controlled by an activator/repressor, synchronized lysis circuit (Din et al., 2016).

### Transport and release of cells

**Figure 3:**
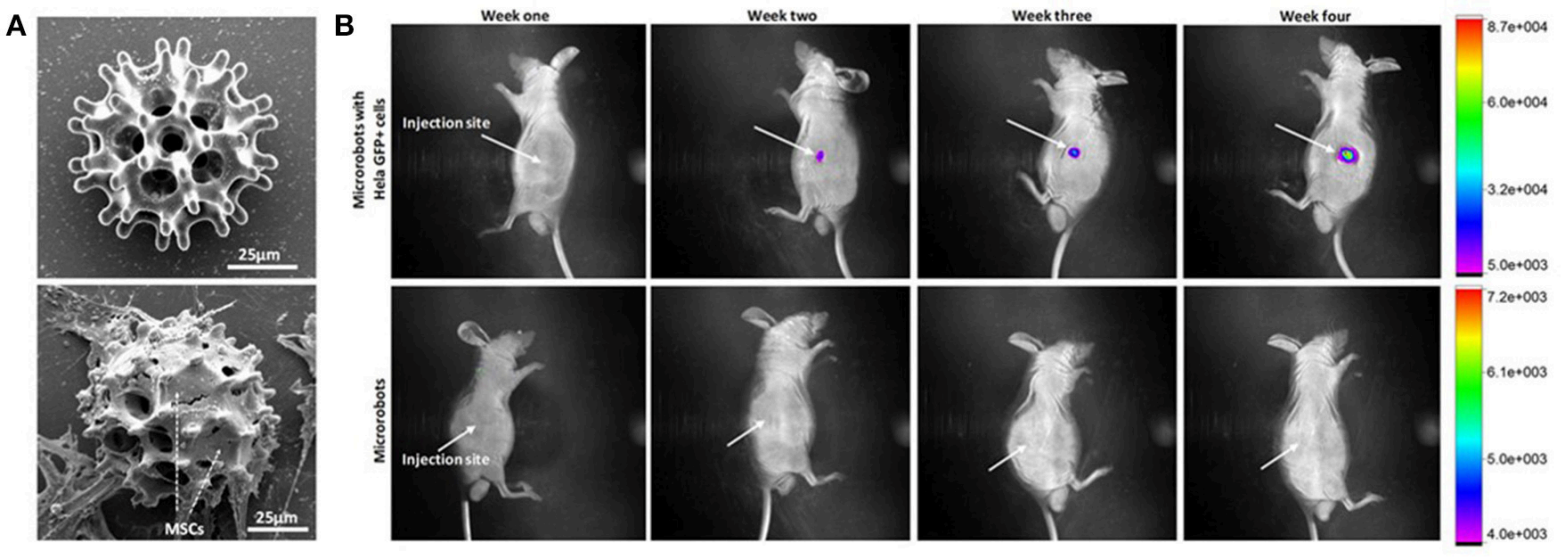
Use of microrobots for cell transport and proliferation of cells. **(A)** SEM images of magnetically actuated nanorobot before and after cell seeding. **(B)** *In vivo* fluorescence imaging of HeLa GFP with cells loaded nanorobots illustrating the migration of cells after injection into the right dorsum of the nude mice (Li et al., 2018). Copyright 2018 The American Association for the Advancement of Science.

Micro/nanorobots have also been used toward delivering stem cells to a damaged location for tissue restoration. Magnetically guided microrobots have been reported toward carrying and delivering live cells to targeted areas in the body. *In vivo* transport and proliferation of *HeLa* cells in a nude mouse model demonstrated that the carried cells could be spontaneously released from the microrobot to the surrounding tissues and proliferate as shown in Figure 3 (Li et al., 2018). These applications demonstrate that micro/nanorobots could serve as platforms for regenerative medicine and cell-based therapy, potentially proving to be especially useful in the later stages of life, when organs and systems start to fail. Moreover, there are still plenty of other promising *in vitro* applications to be developed for biohybrid microrobots, such as using a helical structure to guide a sperm toward an egg, for assisted fertilization (Magdanz et al., 2017).

### Retention of payloads in the gastrointestinal tract

The aim of medical micro/nanorobotics is not only to deliver therapeutic payloads to a specific site but also, to retain the payloads within site as long as possible. In this direction, Wang's group has proposed the use of biodegradable zinc and magnesium powered microrobots that utilize gastric and intestinal fluids as fuels to promote cargo retention in the stomach and intestinal tissues (Gao et al., 2015; Esteban-Fernández de Ávila et al., 2017a). This retention platforms have been applied toward pH neutralization of the gastric fluid (Li et al., 2017a) and for the treatment of a bacterial infection (*Helicobacter pylori*) in the stomach (Esteban-Fernández de Ávila et al., 2017b). This retention of the microrobot could be explained by direct piercing the surrounding tissue, or by an improvement in mass transport and nucleation due to the gas bubbles generated as means of locomotion for the microrobot, in an effect similar to effervescence.

**Figure 4:**
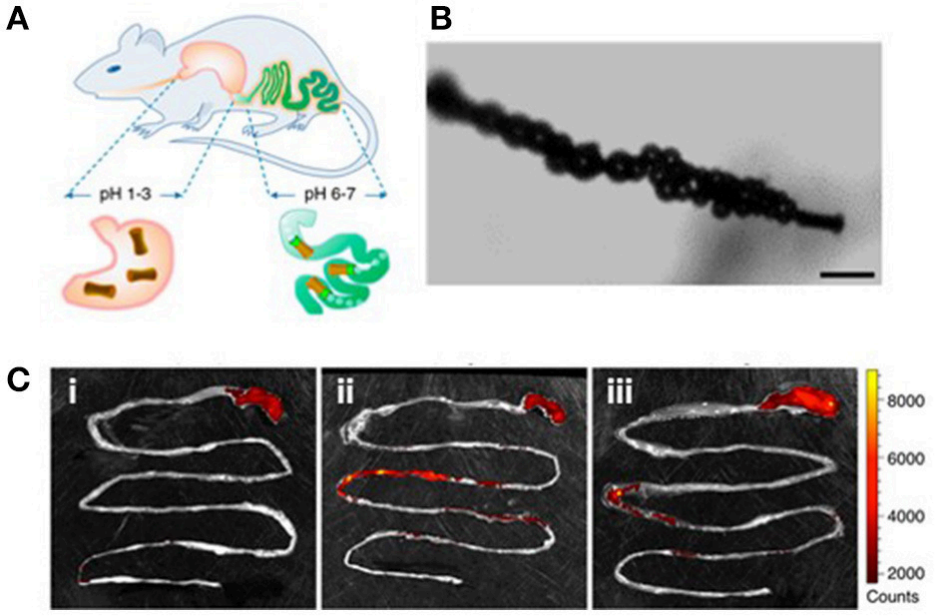
Use of microrobots for enhanced retention of payloads in the gastrointestinal tract. **(A)** Schematic illustration of *in vivo* operation of microrobots in mouse model. **(B)** Micrograph illustrating the bubble generation at the end of the microorobot responsible for locomotion. Scale bar: 20 μm. **(C)** Fluorescent images illustrating the gastrointestinal track retention of the dye Rhodamine 6G delivered by the chemically propelled microrobot (i: control, ii: after 6 h, and iii: after 12 h of administration. Reprinted with permission from Li et al. (2016). Copyright 2016 American Chemical Society.

Magnesium-based microrobots have also been designed with built-in delay activation, by using polymeric enteric coatings that activate the microrobot motion based on their thickness or environmental pH conditions. The polymeric coating only dissolves at neutral pH conditions found in the intestinal fluids resulting in localized ignition of the microrobot. The thickness of the coating allows to selectively localize the retention of the microrobots in different target sections of the gastrointestinal tract (the duodenum, jejunum, and ileum) (Figure 4) (Li et al., 2016). More recently, microrobots were integrated/loaded inside a pill matrix toward streamlining their administration with existing pharmaceutical protocols (Karshalev et al., 2018). In general, the use of magnesium-based microrobots could benefit medical applications where autonomy and simplicity are desired. However, these chemically-propelled microrobots might be limited to operate in large-scale areas, such as the digestive system, as their depletion of propellant through bubble generation results in a short lifetime that could create unexpected complications in smaller capillaries.

### Wound healing

The human body has diverse mechanisms and biological triggers for identifying wounds and repairing them. Nevertheless, these biological mechanisms can fall short when the wound is bleeding profusely, or there are not enough localized coagulant agents in the target region (Das and Baker, 2016). In this direction, medical micro/nanorobotics aim to simulate such systems by using active delivery toward fast and effective wound healing. Chemically-propelled calcium carbonate-based microrobots have been reported for delivering thrombin to halt the bleeding of wounds in the vasculature of mouse and pig models. The distribution mechanism relied on a combination of lateral propulsion, buoyant rise and convection (Baylis et al., 2015). Another reported approach consisted in the use of locomotive microrobots toward laser-based wound sealing. The high temperature generated by the laser-microrobot interaction produced localized collagen denaturation and melting, whereby a subsequent temperature decrease allowed condensation and wound closure (He et al., 2016).

### Biopsy

Other *in vivo* applications with potential implications in medicine are the ones targeting biopsy/surgery. Diverse *in vitro* platforms have been proposed toward precision micro/nanoscale surgery but still have not been translated to *in vivo* models (Nelson et al., 2010; Xi et al., 2013; Kwan et al., 2015; Soto et al., 2015). Nonetheless, micro/nanorobotics could serve as a complement to current minimally surgical procedures, allowing unprecedented access into diseased tissues for biopsy analysis or therapeutic applications.

Microrobots with star-shaped grippers, which can reach narrow conduits in the body, have been used to excise tissue samples from a pig bile duct (Gultepe et al., 2012). Additionally, an initial proof of concept based on magnetic microrobots has demonstrated controlled navigation inside the eye of a living rabbit. Although this method has not been demonstrated directly for a surgical procedure, a magnetic coil system enabled the precise navigation of the untethered magnetic microrobots in the posterior eye section (Ullrich et al., 2013; Pokki et al., 2016). Ultimately, biopsy applications are fertile ground for further micro/nanorobotics research as the success of these applications depends on the ability of the robot to physically manipulate its environment and on the ability of the robot's controller to retrieve the robot. Both these problems have been studied much less extensively than propulsion.

### Local mixing for enhanced thrombolysis

The potential modes through which a micro/nanorobot can physically manipulate its environment are not limited to merely excising tissue. Another recent trend in *in vivo* micro/nanorobots applications is the use of mixing effects to promote blood clot dissolution. In this case, magnetically actuated nanorobots loaded with tissue plasminogen activator were intravenously injected. The vasculature flow drove the nanorobots to the blood clot. Once at their destination, the nanorobots were rotated by an external magnetic field. Their rotation generated local flow mixing which induces an increased interaction of the tissue plasminogen activator molecule with the blood clot interface, resulting in acceleration of thrombolysis (Cheng et al., 2014). More recently, it was demonstrated that nanorobots can target blood clots in mice's brains (Hu et al., 2018).

### Real-time imaging

**Figure 5:**
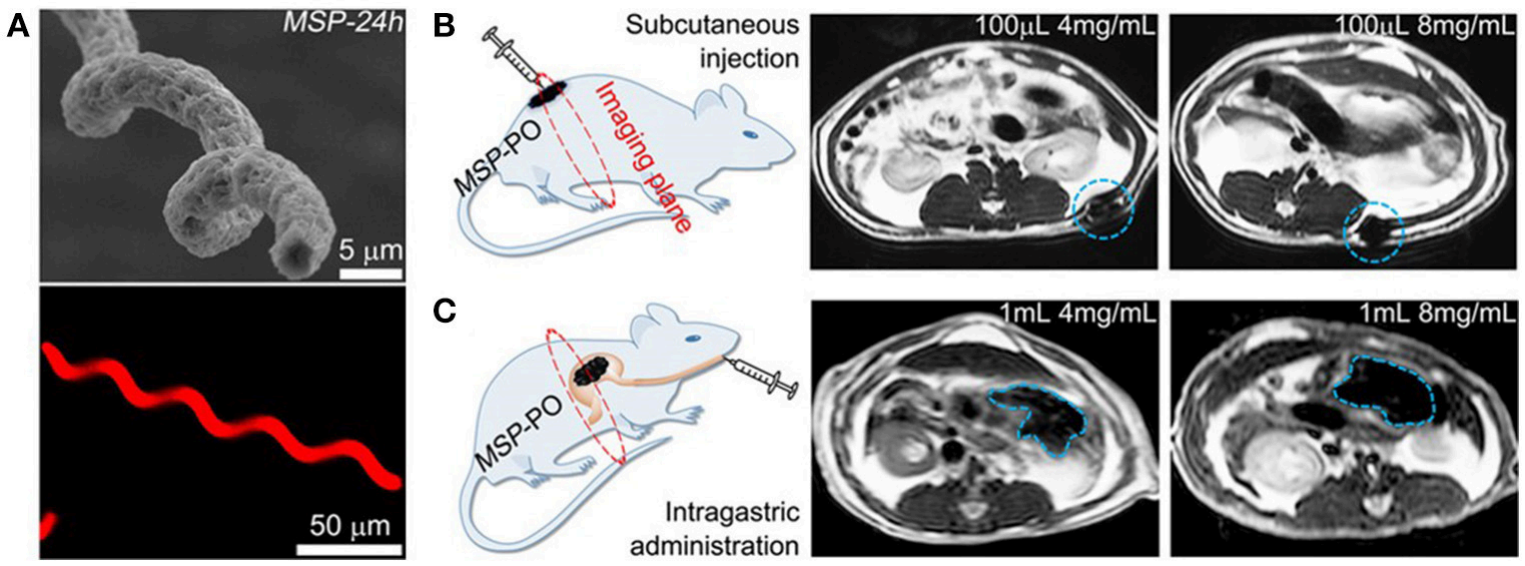
*In vivo* imaging of magnetically propelled microrobot. **(A)** Scanning electron microscopy (SEM) (**top**) and fluorescence images (**bottom**) of the helical structured microrobot. Schematic of the target *in vivo* area and magnetic resonance imaging of microrobots inside rats. Illustrating different microrobot concentrations at the **(B)** subcutaneous tissues and **(C)** inside the mouse stomach. Reprinted with permission from Yan et al. (2017). Copyright 2017 The American Association for the Advancement of Science.

From the reviewed articles, none of the chemically propelled micro/nanorobots are supported with real-time imaging, which introduces a severe limitation for understanding their therapeutic effect. 75% of biohybrid robots are supported with real-time imaging, but fluorescence is the only technique used (Akin et al., 2007; Park et al., 2013; Din et al., 2016). Physical robots are supported with the most diverse type of imaging techniques, but only 60% of articles are supported with real-time imaging. Represented techniques include the use of endoscopy and X-rays to detect microgrippers inside the gastrointestinal tract (Gultepe et al., 2012), use of optical camera to visualize movement inside the eye (Ullrich et al., 2013; Pokki et al., 2016), and fluorescence imaging techniques to track the position of magnetically actuated helical microrobots inside the peritoneal cavity of a mouse (Servant et al., 2015) or subcutaneously (Li et al., 2018). Moreover, a dual imaging approach was used to detect biodegradable magnetic microhelix nanorobots in mice. Fluorescence imaging was used to detect the nanorobot's position inside the subcutaneous tissue, and the intraperitoneal cavity of a mouse and magnetic resonance-based imaging was used to detect the nanorobot's position inside the mouse's stomach, as shown in Figure 5 (Yan et al., 2017). Future research such address this important parameter, a key aspect for medical micro/nanorobotic use in clinical uses will rely on individual or population tracking, with consideration of tissue background signal (Medina-Sánchez and Schmidt, 2017; Vilela et al., 2018; Wang et al., 2018a).

### Toxicity

Most of the review micro/nanorobot studies used in animal models, provide only qualitative analyses of safety and toxicology based on histological assays. Achieving accurate targeted delivery requires an understanding of how foreign materials accumulate throughout the body and how to minimize the distribution of the administered micro/nanorobots to non-target tissue, establishing their specific effect on health. Each micro/nanorobot design has different safety concerns. Biohybrids could infiltrate and proliferate in undesired ways. Chemically propelled micro/nanorobots might change the local chemical environment which could have a significant effect on the microbiome of the gastrointestinal tract. For physical micro/nanorobots, most of the materials themselves pose a danger, as they are rigid and non-degradable. Despite these differences, researchers could report more relevant parameters to address the toxicity even at this early stage. Potential parameters to be considered include: the number of motors used for treatment (number or grams) indicated the escalation of units/dosage (limit for toxicity and inefficacy), but most importantly the distribution and toxicity of the nanorobot's constitutive materials within the living system.

### Administration and retrieval

The administration and retrieval strategy of nanorobots is not discussed in length in the reviewed articles, which present as the most common administration method injection (60%), followed by oral administration (30%), catheter (5%), and topical administration (5%). Regarding the retrieval strategy for micro/nanorobots, both biohybrid and chemical systems were considered to be biodegradable by the authors. Although, in some cases, the fate of their synthetic components was not explicitly stated or demonstrated. For physically powered robots, in most cases, the fate of the nanorobot structure was not clearly stated, only three articles provide a retrieval mechanism and one other is biodegradable. One plausible solution for retrieval consists of the use of a magnetic catheter to enable both the deployment and retrieval of microrobots in clinical practice (Iacovacci et al., 2018). We note that recent *in vitro* research efforts have described micro/nanorobot systems that are fully biodegradable (Peters et al., 2015; Chen et al., 2016; Yan et al., 2017; Bozuyuk et al., 2018; Wang et al., 2018c).

## Micro/nanorobots: steps toward clinical translation

Basic science research is fundamental for creating both breakthrough medical advances and economic growth. Nevertheless, most basic science research will not achieve clinical translation. This challenge arises, in part, from the prolonged time required to go from basic research to clinical trials. Moreover, the conclusion of most trials indicates that most of the new technologies are less effective or more harmful than the current standards of care. This is a lesson to be learned in the micro/nanorobotic community. It is essential to identify strengths/weaknesses and remain objective about their relevance for later development.

The potential of the current generation of micro/nanorobotics is based on their translational motion and mixing capabilities. The idea of “smart” nanorobots that explore their surroundings and respond to environmental stimuli, commonly described in the field, is still beyond the current capabilities of micro/nanorobotics. Crucial questions of environmental manipulation, micro/nanorobot retrieval, and toxicology still need to be addressed. Indeed, we are far away from a killer app, but this should not be considered uncommon or discouraging to current researchers in the field. As previously mentioned, medical technologies have a significant time delay from lab to the clinic/commercialization, in many cases, taking several decades.

Nevertheless, considerable progress has been achieved in the field. It took less than a decade to go from the initial proof of concept using chemically propelled nanowires in peroxide (Paxton et al., 2004) to the recent explosion of *in vivo* studies over the past 3 years. In order to reach the inflection point toward clinical translation, it is necessary first to standardize the methodologies and reproducibility of micro/nanorobotics research and to actively consider their commercialization outlook. Scientists without clear monetary value proposition rarely see their discoveries materialize.

### Reproducibility and standardization

Commonly, the performance of micro/nanorobotic medical technology *in vitro* is reported by measurements of movement, such as their velocity and directionality, or on indirect methods such as measuring the mean squared displacement of tracer particles or comparing a clinical outcome (e.g., the percentage of dead cancer cells) vs. passive diffusion. Although these methods serve to validate early proof-of-concept studies, they are not good predictors for successful clinical translation.

The variability between currently used *in vivo* micro/nanorobotic platforms makes it almost impossible to provide a comparative or quantifiable analysis of their efficiency vs. current standards of care. Moreover, there cannot be a clear definition of clinical efficiency when there is no minimum standardized information for reporting micro/nanorobotic research. Therefore, we need clear information standards in reporting experimental methodology, characterization, and results, which will be key for the successful translation of multidisciplinary research into improved health outcomes.

Some journals have implemented reporting summaries as a requisite for submission (Faria et al., 2018), which has shown to increase the quality of reporting for preclinical biomedical research (Han et al., 2017). In general, an experimental finding can be considered to have a stronger case for translation when the experiments supporting it have been carried out using blinding and randomization, and when, most importantly, those findings have been replicated in other laboratories apart from the inventors' group. Currently, no study presented in this review covers these standards. In this direction, here we suggest guidelines (measures that should be reported in parenthesis) for reporting micro/nanorobotic research that should allow better comparisons, both qualitative and quantitative, of micro/nanorobot performance in addition to already established checklists for safety and toxicology. The goal of the checklist is not to propose research trends or criticize current research, as new technologies start without clear standards. Instead, the homogeneity of experimental reporting can facilitate comparison, improve experimental design, and increase reproducibility between various micro/nanorobotic platforms.

The justification of the power source used to provide energy/movement to the micro/nanorobot could help to establish the unique advantage of each method. Material characterization of the micro/nanorobot, including their size distribution (length units), average lifetime of propulsion (time unit), surface charge (zeta potential), and storage lifetime (days), can help to plan into eventual fabrication and transport in medical uses. Moreover, is important to quantify the performance of the nanorobotic platform by evaluating the therapeutic effect based on experiment dependent parameters. For drug delivery applications, an important parameter is the administered and delivered dose (% by mass). For retention studies the number of nanomotors localized in the target region (% of administered population). For biopsy applications, the micro/nanorobot force applied to a specific area of tissue (Pascal). Moreover, the micro/nanorobot therapeutic effect should be compared against the state-of-the-art treatment, not only passive diffusion. Finally, parameters covered through the review includes, imaging (real time localization), toxicity (folllowing establish guidelines), Administration route (Target Area and method of insertion), and retrival region (% of administered population) could streamline their tranlatio into exisitng therapies.

### Intellectual property and commercialization

Clinical efficacy alone is not enough to warrant translation to market. The transition of new technologies from lab to market requires extensive research and development costs, overcoming regulatory barriers, and most importantly, having the right commercialization potential. The potential customer for micro/nanorobotics' technology is not the general population or the physicians, but rather pharmaceutical companies and insurance providers. Therefore, micro/nanorobotics research should consider their commercialization opportunities from the research stages, in order to provide tangible therapies that reduce cost, increase throughput efficiency and productivity.

Minimizing the investment risk can be achieved by protecting the intellectual property of the new technological developments (which is one of the most valuable assets for high-tech companies) and establishing a clear business model with identifiable revenue streams. New technological developments are commonly initiated using patents filed by research groups through their university system, which are later licensed to emerging start-up companies or larger corporations. The prohibitive cost of prototyping and clinical trials is initially financed by non-dilute financing, which includes government research and industry grants, and at later stages by investment from venture capital and industry partnerships.

**Figure 6:**
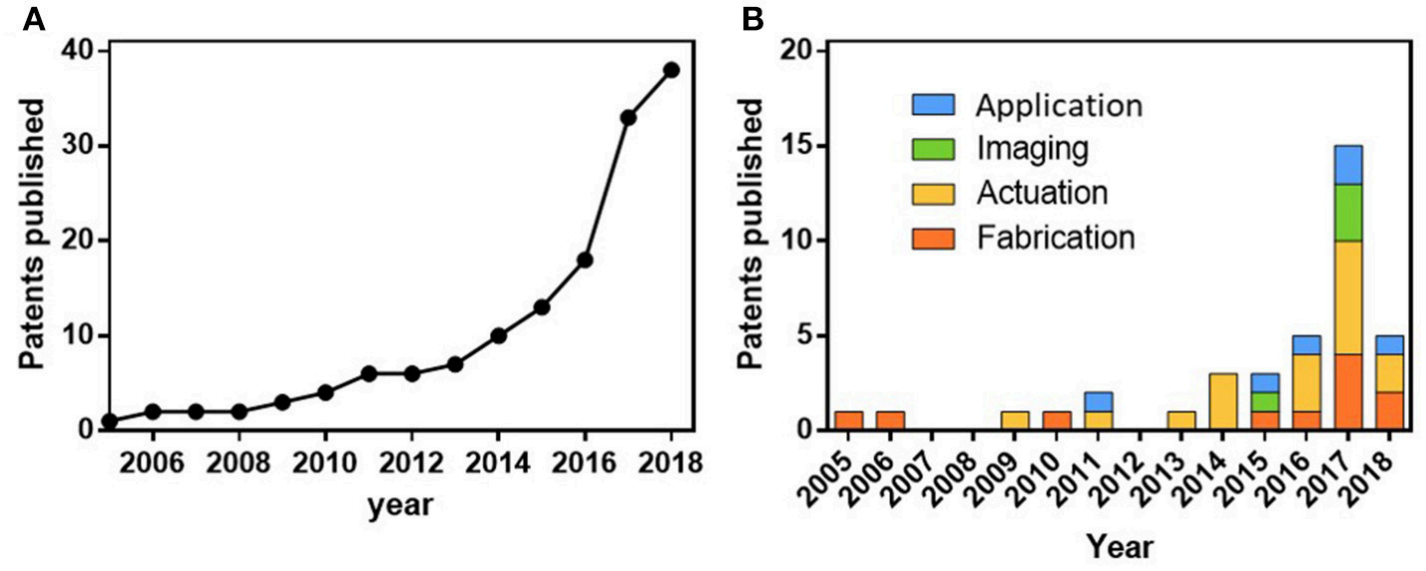
Overview of micro/nanorobotics intellectual property, as described in section Intellectual Property and Commercialization. **(A)** Accumulative published patents in the last years. **(B)** Graph illustrating patents considering the application per year.

Tracking the filing of relevant patents, and the formation of relevant startups, provides useful insight into the progress made in identifying potential markets, competitors and consumer needs. Figure 6 traces the accumulated United States applied and granted patents in nanorobotics, while also separating them into the categories of fabrication, actuation, imaging, and application. The patent filing data were collected manually, as keyword searches do not accurately capture the state of nanorobotic technology due to the multiple names used to refer to nanorobots in the literature (micro/nanobot, micro/nanorobot, micro/nanorocket, micro/nanoengines, micro/nanomotor, micro/nano-swimmer, active colloid). Instead, the search was conducted by reviewing the output of the most prolific nanomotor research groups in the field that have published results in animal models and by reviewing relevant Google patents.

The first patents published consisted of the fabrication methods commonly used for parallel mass fabrication using template platforms, consisting of nanorod structures (Stonas et al., 2005; Natan et al., 2007; Fan et al., 2017b; Odell et al., 2018), microcoils (Schmidt et al., 2010; Jeong et al., 2018), tubular structures (Yao et al., 2015; Wang et al., 2016a), emulsions (Percec et al., 2017), biohybrids (Schmidt and Leibniz-Institut Fuer Festkoerper-Und Werkstofffor Schung, 2017), and colloid chains (Duan et al., 2017).

The next wave of patents consists of tracking/imaging nanorobots using magnetic (Martel et al., 2015; Muntwyler et al., 2017; Odell et al., 2017), optical (Benaron et al., 2008), and ultrasound methods (Mattrey et al., 2017). Followed by methods to power micro/nanorobots using magnetic (Solomon and Solomon Res LLC, 2011; Vollmers et al., 2013; Abbott et al., 2014; Fischer et al., 2014; Mahoney et al., 2014; Odell et al., 2016; Sitti et al., 2016; Tasci et al., 2016; Wang, 2017; Weinberg et al., 2017), ultrasound (Wang and Zhang, 2017; Wang et al., 2017), chemical (Paxton et al., 2009; Sen et al., 2017; Tang et al., 2017), and biohybrid propulsion mechanisms (Magdanz et al., 2018; Martel et al., 2018).

The final, most recent wave, has focused on applications for these micro/nanorobots, including for intracellular delivery by nanospearing (Cai et al., 2011) exploration of subterranean geophysical formations and oil retrieval (Kamal et al., 2015), nanomotor-based patterning of surface microstructures (Wang et al., 2016b), transporting, positioning and assembling nanorobots using electric twisers (Chien et al., 2017; Fan et al., 2017a), capturing and isolating of target biomolecules and living organisms using microrobots (Wang et al., 2018b).

There are only a few active companies working toward the commercialization of nanorobots for use in medical applications. Most notably, Nelson's group has spun off two companies. The first was Aeon Scientific, based on the electromagnetic manipulations systems developed to guide magnetic motors. However, they have recently shifted their focus to developing new manipulation systems for catheters. Indeed, it is not surprising that the technology developed around micro/nanorobots could have applications that were never envisioned at the beginning.

The second, Swiss Magnetibox formed in 2014 is based on wireless tools for actuating and imaging micro/nanomachines using magnetic fields. They primarily sell microscopy equipment coupled with magnetic actuation systems and rod shape microrobots toward expanding basic science in micro/nano robotics research. Although these systems are not specifically designed for *in vivo* use, they provide the necessary tools to test proof of concept applications (Schuerle et al., 2017).

Finally, Weingberg medical physics has developed an image-guided therapy for targeted delivery of magnetic micro/nanorobots that uses ultra-fast MRI to generate magnetic fields for imaging and manipulation of the magnetic nanorobots. They have demonstrated diverse *in vitro* applications using the mechanical force of the nanorobots to dislodge bacteria biofilm using rotating nanorobots (Mair et al., 2017) and drilling into mice's brain post-mortem (Jafari et al., 2019).

The mass fabrication of nanorobotics is one of the first challenges to be addressed toward tangible translation to market. Reproducibility and availability of templates will be significant hurdles that must be solved with traditional micro/nanorobotic fabrication techniques. New methods based on 3D printing and two-photon lithography could allow for complex designs such as microhelices or cell carriers. Although, the cost and slow production throughput of these methods may limit the use of such intricate designs. Moreover, new methods of characterization and manipulation of individual nanorobots are necessary to ensure the quality of each batch. In this regard, nano-manipulation systems are ideal for transporting, inspecting and testing nanorobotic designs (Wang et al., 2015; Lu et al., 2017; Meng et al., 2017; Zhang et al., 2018). Once these technical capabilities are well established, the reduction in the cost to reach the market will allow new companies to spring up and provide innovative medical value propositions.

A possible path toward early adoption of micro/nanorobots in the clinic could be achieved by offering micro/nanorobotic technology as a complementary tool to existing medical procedures. For example, their integration with current oral delivery platforms such as pills, allow for dose escalation by increased retention of therapeutic payloads at the mucosae wall. Although special consideration should be placed in therapies where the primary therapeutic effect is based on systemic distribution, as in this case, the dose escalation proposed by micro/nanorobotic platforms is not desired. Moreover, micro/nanorobots used for tissue biopsy and suturing veins could be integrated with a catheter and mili surgical robotics, allowing the larger robots to reach scale ranges where their size normally does not permit them to operate. The possibilities discussed here are driven by the need to improve patients' outcomes after surgical procedures including reduced hospital stay, lower chances of infection, and minimum scarring. We should note that it is not likely that micro/nanorobots will be used for preventive care or as a chronic treatment since the sustained introduction of synthetic objects into the human body might produce unknown repercussion due to possible accumulation.

## Future outlook

The field of medical nanorobotics has achieved considerable advances. However, several issues and challenges must be addressed before micro/nanorobots could have real-world clinical applications. The goal of the *in vivo* model is not only to evaluate the therapeutic efficiency of the platforms, but to identify the clinical risk, as evaluating off-target effects of nanorobots is as essential as evaluating efficacy. Indeed, there is a discrepancy between the aspirations of medical nanorobotics and reality, as the legacy of science fiction has set the conceptual boundaries of what to expect long before scientists could. The manufacturing of the micro/nanostructure engines must be optimized, with special consideration for material biocompatibility and degradation, to address *in vivo* safety concerns.

Furthermore, proper standards should be established to clarify the advantages of micro/nanorobot therapies over traditional methods which already fulfill FDA standards. Nonetheless, micro/nanorobotics might potentially improve medical diagnosis and treatment. We should also consider that the designs and aspirations of a small group of scientist and engineers could soon affect the lives of millions of people directly and profoundly, therefore it is essential to consider the economic, social, and ethical implications of the use of medical nanorobotics. These implications are likely to be on par with those of the most significant technological revolutions.

## Author contributions

FS and RC wrote and revised the manuscript and approved it for publication.

### Conflict of interest statement

The authors declare that the research was conducted in the absence of any commercial or financial relationships that could be construed as a potential conflict of interest.
